# Experimental Inoculation of Porcine Circovirus 3 (PCV-3) in Pregnant Gilts Causes PCV-3-Associated Lesions in Newborn Piglets that Persist until Weaning

**DOI:** 10.1155/2023/5270254

**Published:** 2023-10-20

**Authors:** Àlex Cobos, Albert Ruiz, Mónica Pérez, Anna Llorens, Eva Huerta, Florencia Correa-Fiz, Robert Lohse, Mònica Balasch, Joaquim Segalés, Marina Sibila

**Affiliations:** ^1^Unitat Mixta d'Investigació IRTA-UAB en Sanitat Animal, Centre de Recerca en Sanitat Animal (CReSA), Campus de la Universitat Autònoma de Barcelona (UAB), Bellaterra, Barcelona 08193, Catalonia, Spain; ^2^Departament de Sanitat i Anatomia Animals, Facultat de Veterinària, Campus de la Universitat Autònoma de Barcelona (UAB), Bellaterra, Barcelona 08193, Catalonia, Spain; ^3^WOAH Collaborating Centre for the Research and Control of Emerging and Re-Emerging Swine Diseases in Europe (IRTA-CReSA), Bellaterra, Barcelona, Spain; ^4^IRTA Programa de Sanitat Animal, Centre de Recerca en Sanitat Animal (CReSA), Campus de la Universitat Autònoma de Barcelona (UAB), Bellaterra, Barcelona 08193, Catalonia, Spain; ^5^Zoetis Manufacturing and Research Spain S.L., Ctra. Camprodon s/n La Riba, 17813 Vall de Bianya, Girona, Spain; ^6^Zoetis Inc., 333 Portage Street, Kalamazoo 49007, MI, USA

## Abstract

Porcine circovirus 3 (PCV-3) has been detected in cases of reproductive failure but the pathogenesis of such infection is poorly understood. So far, experimental PCV-3 inoculations have been performed only in piglets. Therefore, through the experimental inoculation of pregnant gilts at two different time points (second and last third of gestation), this study aimed to evaluate the outcome of PCV-3 infection in dams and their offspring until weaning age. Two weeks postinoculation, all gilts became viremic and the infection lasted until the end of study. Farrowing occurred naturally, without evidence of reproductive disorders, and piglets showed no significant clinical signs from farrowing to weaning (21 day-old). However, majority of the delivered piglets were viremic, mostly until weaning age. Both newborn and weaned pigs showed different degrees of systemic, lymphohistiocytic arteritis and periarteritis. Lesions were more severe in the piglets infected during the second third of gestation and worsened at weaning. Additionally, PCV-3 detection in nervous and cardiac tissue and development of histopathological lesions in these tissues were gestational dependent, as only occurred in piglets infected at second third of pregnancy. Piglets with lesions raised to weaning age had less body weight than those without them. This study represents the first description of a PCV-3 experimental infection in pregnant gilts, which resulted in transplacental infection, histological lesions in piglets mimicking those of natural occurring disease, and lesser body weight in piglets with vascular lesions at weaning age. Obtained results allowed proposing a potential pathogenesis model for PCV-3 infection in swine.

## 1. Introduction

Porcine circovirus 3 (PCV-3) was first discovered by metagenomics in sows displaying reproductive failure and porcine dermatitis and nephropathy syndrome (PDNS)-like signs [1]. Epidemiologically, viral DNA has been detected with high prevalence in pigs of different ages without apparent clinical signs, both in prospective [2–4] and retrospective [5] studies. These data raised concern about its pathogenic potential and overall role in swine industry as a pathogen.

The detection of PCV-3 in microscopic lesions of sick pigs has prompted to suggest the establishment of disease case definition and diagnostic criteria [6] for two presumably independent diseases outcomes. First, PCV-3 reproductive disease (PCV-3-RD), mainly characterized by an increased number of stillborn and mummified fetuses and weak born piglets, which may die few days after birth [1, 7–9]. Second, PCV-3 can be found in postnatal pigs showing growth retardation, wasting, and anorexia [9–11], condition that has been named as PCV-3 systemic disease (PCV-3-SD). Both PCV-3-RD and PCV-3-SD have similar proposed diagnostic criteria consisting of compatible clinical signs, characteristic histologic lesions (multisystemic inflammation in—but not limited to—arteries), and detection of moderate to high amount of viral genome by in situ hybridization (ISH) in these inflamed tissues. Cases fulfilling these criteria have been detected in samples from sick piglets prospectively and—more importantly—in retrospective studies [10], which evidence that PCV-3 had not only been circulating subclinically for decades but also causing disease. Besides PCV-3-RD and PCV-3-SD, cases of congenital tremors (CT) and PDNS have been associated with PCV-3 infection [1, 12]. However, more recent prospective [13] and retrospective [10] studies did not support such relationship.

To better understand PCV-3 overall impact in swine health, efforts to understand its pathogenesis are mandatory. Since PCV-3 discovery, few experimental infections have been performed, probably due to the difficulties to isolate the virus [14]. Experimental inoculations published so far targeted young piglets, with variable success in reproducing histological lesions as seen in natural cases [15–17]. Additionally, one of these experimental inoculations resulted in multisystemic inflammation that was classified as PDNS by the authors [18], despite the typical necrotizing arteritis and fibrino-necrotizing glomerulonephritis of this condition [19] were not reported.

To the authors' knowledge, reproduction of PCV-3-RD has not been attempted at experimental level, even though reproductive disorders seem to be an important outcome of PCV-3 infection [6]. Viral detection in fetal samples revealed higher percentage of infection in fetuses of the last third of gestation, although few ones were also quantitative PCR (qPCR) positive in the second third of pregnancy [20]. Therefore, it seems clear that PCV-3 can infect fetuses at different time points of the gestation. However, whether the specific infection timing during pregnancy plays a role in disease outcome or not is unknown. Consequently, the objective of this study was to inoculate pregnant gilts with PCV-3 at different gestational moments to evaluate the reproductive and pathologic outcomes, trying to establish a likely pathogenetic model for this viral infection.

## 2. Materials and Methods

### 2.1. Ethics Statement

This study protocol (PJ106) was reviewed and approved by the Zoetis Ethical Review Board and was approved by the Ethics Committee of the regulatory authorities (No. 11368 of the Catalan *Departament d'Acció Climàtica*, *Alimentació i Agenda Rural*) according to Spanish law.

### 2.2. PCV-3 Isolate

A PCV-3 isolate (PCV3/USA/IA/ISU44806/2018) propagated and isolated in PK-15 cells (ATCC CCL-33; *Sus scrofa*; kidney; USA) at Iowa State University [16] was used as inoculum. Briefly, after propagation in Eagle's Minimum Essential Medium (EMEM), cells were lysed by three cycles of freezing and thawing, obtaining the challenge material, which was approximately 10^5^ 50% tissue culture infective dose per mL (TCID_50_/mL). The inoculum was stored at −80 ± 10°C. Healthy PCV-3-free PK-15 cells cultured with EMEM were lysed with the same procedure, which was then used as mock inoculum.

### 2.3. Animal Challenge

A total of 13 pregnant gilts (7–9 months of age) were included in the study and randomly distributed among three groups. The gilts were housed in biocontainment facilities, isolated by treatment group, and were clinically healthy, as well as negative to PCV-2 qPCR (VetMaxTM Porcine PCV2 Quant Kit, Applied Biosystems, Lissieu, France) and PCV-3 qPCR [8] in serum samples at the beginning of gestation (when pregnancy was confirmed by echography). Gilts were serologically negative against porcine respiratory and reproductive syndrome virus (PRRSV) (IDEXX PRRS X3 Kit, IDEXX Liebefeld-Bern, Switzerland) but resulted positive against porcine parvovirus (PPV) (INgezim PPV Kit, Ingenasa, Madrid, Spain). Since no antibody tests are validated and available for PCV-3, the serological status of gilts for this virus was unknown.

Gilts from group T1 (*n* = 6) were inoculated at study day (SD) 0 (39–51 days of gestation, DOG) and gilts from group T2 (*n* = 5) were inoculated at SD 35 (75–86 DOG) with a dose of the PCV-3 isolate by intranasal (4 mL) and intramuscular (2 mL) routes to maximize the likelihood of infection. Since immunocompetence in pig fetuses is achieved around 70 days of gestation [21], the timing of inoculations represented the eventual infection of fetuses in preimmunocompetence (T1 group) or postimmunocompetence (T2 group) stage. A T3 group composed of two pregnant gilts served as negative control. Mock inoculations were performed (T2 and T3 at SD 0 and T1 and T3 at SD 35) with the same route and volume of EMEM.

Rectal temperature of gilts was recorded every other day for 10 days after both inoculations, or, when present, after fever resolved (fever was considered when rectal temperature was above 40.5°C). Clinical observations were recorded weekly. Blood samples were collected from gilts at SD 0 and weekly thereafter. Gilts were allowed farrowing naturally, and health status and reproductive parameters (numbers of born alive, stillborns, and mummies) were recorded. Approximately half of the piglets per litter were euthanized with an intravenous injection of an overdose of sodium pentobarbital (>100 mg/kg) within the day of delivery. The other half of the piglets were followed-up until weaning (19–31 days of age). During the lactating period, health status of the piglets was monitored three times a week. Body weight was recorded from all piglets at farrowing (SD 64–76) and at weaning (SD 92–95), and the average daily weight gain (ADWG) was calculated from birth to weaning. At weaning, the remaining piglets were euthanized with the same procedure indicated above, and gilts were sedated with a combination of tiletamine, zolazepam, xylazine, and ketamine 50 mg/mL at 5 mL/100 kg by IM route and then euthanized with T61® (embutramide 200 mg/mL, mebezonium iodide 50 mg/mL, and tetracaine hydrochloride 5 mg/mL) administered 4–6 mL/50 kg by IV route.

### 2.4. Postmortem Examination

All euthanized animals (both gilts and piglets) were subjected to postmortem macroscopic examination and tissue and blood sampling. Blood was centrifuged at 860 *g* and sera stored at −80 ± 10°C until used. A subset of tissues (tonsil, tracheobronchial lymph node, mesenteric lymph node, superficial inguinal lymph node, brain, lung, heart, kidney, liver, and spleen) were collected and fixed in 10% buffered formalin for histopathological analysis. Some of these tissues (mesenteric lymph node, brain, lung, heart, and spleen) were also kept frozen at −80 ± 10°C for further PCV-3 qPCR analyses. If present, mummified and stillbirths were also necropsied and the same tissue samples were collected for histopathological and qPCR analyses.

### 2.5. Real-Time PCR from Serum and Tissues

Fresh tissue samples were macerated individually using a tissue lyser. Afterward, 40 *µ*L of each of the five macerated tissues per animal were pooled. Serum and pooled tissue macerates were processed by qPCR to detect and quantify the presence of PCV-3 genome. DNA extraction from sera and tissue homogenates was done using the MagMAX^TM^ Pathogen RNA/DNA Kit (Applied Biosystems®, Vilnius, Lithuania), following the manufacturer's protocol. The qPCR analysis was performed targeting PCV-3 as previously described [8]. Results were expressed as log10 PCV-3 copies/mL of serum or mL of supernatant of tissue homogenate. Samples with a viral load below 3 log10 PCV-3 copies/mL of serum or tissue homogenate were considered positive but not quantifiable (PNQ). In those cases, a viral load of 2.7 log10 was considered (half of the quantification limit, set at 3 log10 PCV-3 copies/mL).

Serum samples from gilts at SD 0 were further processed by qPCR to detect PCV-2 genome (LSI VetMAXTM Porcine PCV2 Quantification Kit, Thermo Fisher, Lissieu, France).

### 2.6. Histopathological Evaluation and PCV-3 in In Situ Hybridization (ISH) of Gilts and a Subset of Selected Piglets

Formalin-fixed tissues of all gilts and piglets were dehydrated in series of alcohol solutions and were embedded in paraffin. Sections of approximately 4 *µ*m thick were obtained from the formalin-fixed and paraffin-embedded (FFPE) tissues and routinely stained with hematoxylin and eosin (H&E) for histopathological evaluation.

The severity of the systemic, lymphohistiocytic arteritis/periarteritis (from now on, periarteritis) lesions was graded only in kidney and mesenteric arteries from 0 to 3 as previously described [10]. In other tissues, presence or absence of periarteritis was recorded. When present, other lesions were also recorded and graded as mild, moderate, or severe (0–3 score) according to the intensity of the inflammatory infiltrates [10].

To further localize and quantify the PCV-3 presence within tissues, a subset of piglets (*n* = 43) were selected based on their PCV-3 load in tissues detected by qPCR: negative, low-intermediate (≤6 log10 genome copies/mL of tissue supernatant), or high amount (>6 log10 genome copies/mL of tissue supernatant). FFPE from these animals was analyzed by ISH using RNAscope technology as previously described [8]. Briefly, an additional 4 *µ*m cut was obtained from the FFPE blocks and deparaffinized. Endogenous peroxidase was blocked, and protease and target retrieval treatments were performed. Then, subsequent incubations with a PCV-3 probe targeting rep gene sequence (catalog no. 491021) and six amplifiers were performed between washes with wash buffer solution, and, finally, signal was detected using red solution. Specificity of labeling was further confirmed using a negative probe (catalog no. 310043). Viral genome amount was graded from 0 to 3 as previously described [10]. Both histological and ISH assessments were conducted by a pathologist in a blinded fashion.

### 2.7. PCV-3 Sequencing

The extracted DNA from the inoculum, as well as from three tissue samples of gilts per group and two from piglets per group, was amplified and sequenced using three different set of primers with Sanger, as previously described [8]. The different amplicons obtained from each sample were processed for quality assessment with FinchTV (FinchTV 1.4.0 (Geospiza, Inc., Seattle, WA, USA; http://www.geospiza.com) software and assembled to obtain the full genome. The cap gene region was extracted, and a multiple sequence alignment was done using the online version of MAFFT version 7 (doi: 10.1093/bib/bbx108). A maximum likelihood phylogenetic tree was built with the best substitution model to inspect the sequence similarity between samples and the inoculum at both nucleotide and amino acid levels.

### 2.8. Statistical Analyses

All data generated across the study were statistically analyzed using the SAS System® version 9.4 (SAS Institute Inc., Cary, NC, USA). The following tests were performed for each variable comparison between study groups at each time point (when applicable):

1. Quantitative variables: Differences between groups were tested using an analysis of variance (ANOVA) if application conditions were satisfied; alternatively, Kruskal–Wallis (KW) test was used. Application conditions were tested using Levene's test for homogeneity of variances and Shapiro–Wilk's test for normality of data distribution for each treatment group.
2. Qualitative variables: Differences between groups were tested using a *χ*^2^ test for homogeneity if application conditions were satisfied (see above); alternatively, likelihood ratio test was used.

To analyze the relationship between two quantitative variables, Pearson or Spearman correlations were calculated. *P*-values for pairwise comparisons were adjusted for multiplicity using Tukey (for ANOVA) or Bonferroni's correction (for KW and *χ*^2^ tests).

All the statistical analyses with the qPCR results were conducted with the log10 of the viral load. The area under the curve (AUC) was calculated with the individual qPCR absolute results and then expressed as the log10.

Graphics for each analysis were created with GraphPad Prism version 9. The symbols in the graphics refer to its statistical significance (absence of asterisk or ns: no significant,  ^*∗*^: *P* < 0.05,  ^*∗*^ ^*∗*^: *P* < 0.01).

## 3. Results

### 3.1. PCV-3 Infection in Gestating Gilts Results in Subclinical Infection and Normal Reproductive Parameters

**Figure 1:**
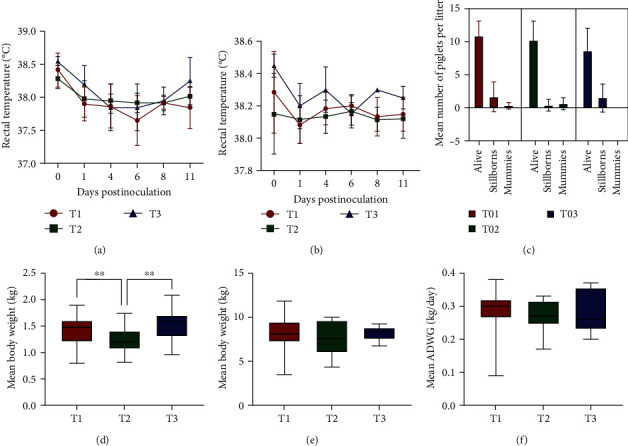
Mean rectal temperature (±standard deviation (SD)) of gilts measured at (a) first challenge (T1 SD 0) and (b) at second challenge (T2, SD 35), and every other day until 10 days postinoculation. (c) Mean number (±SD) of born alive piglets, stillborn and mummified fetuses per litter, for each experimental group. Box plot of mean body weight of piglets at farrowing (d) and at weaning (e) ages for each experimental group. (f) Box plot of mean average daily weight gain (ADWG) of piglets between farrowing and weaning ages for each experimental group ( ^*∗∗*^*P* < 0.01).

All gilts remained clinically healthy across the study. There were no statistically significant differences in the mean rectal temperature between the groups throughout the study (Figures 1(a) and 1(b)).

Farrowing occurred naturally between days 114 and 118 of gestation in all experimental groups. The number of born piglets (including mummified and stillborn) was 77 in T1 (12.8 per gilt), 56 in T2 (11.2 per gilt), and 20 in T3 (10.0 per gilt). The number of stillborn piglets per group was 10/77 (13.0%) for T1, 2/56 (3.5%) for T2, and 3/20 (15.0%) for T3 (Table S1). The number of mummified fetuses per group was 2/77 (3.6%) in T1, 3/56 (5.4%) in T2, and 0/20 (0.0%) in T3. There were no statistically significant differences in the mean number or proportion of alive, stillborn, and mummies per litter across the different groups (Figure 1(c)).

Some of the born alive piglets (3/65; 4.6% in T1 and 3/51; 5.4% in T2) were crushed by the gilt and died shortly after birth (from those piglets, only tissue samples were collected). Additionally, one animal from the T2 group was humanely euthanized at 12 days of age due to poor body condition, anorexia, and prostration; at necropsy, this piglet had suppurative bronchopneumonia.

A total of 89 piglets, including born alive (*n* = 63, randomly selected), crushed (*n* = 6), stillborn (*n* = 15), and mummified (*n* = 5), were necropsied at farrowing, while the remaining 64 were raised to weaning age when they were necropsied. No clinical signs were observed in the piglets throughout the study.

At farrowing, piglets from the T2 group had significantly lower mean body weight than the other two groups (Figure 1(d)). No statistically significant differences on body weight at weaning and ADWG between groups were found (Figures 1(e) and 1(f)).

### 3.2. Infected Gilts Displayed Sustained Viremia Throughout the Study

All gilts included in the study were negative by PCV-2 and PCV-3 qPCRs in serum samples when they were included in the study (pregnancy confirmation) and remained negative at SD 0. Similarly, gilts were seronegative to PRRSV and remained negative at SD 0.

**Figure 2:**
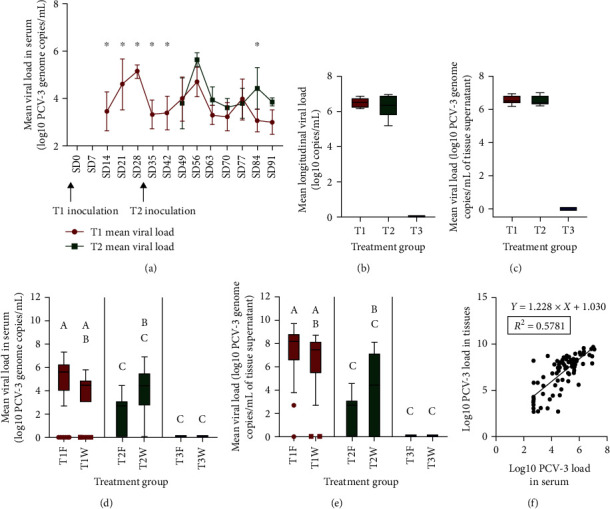
(a) Evolution of mean viral load (±SD) in gilt serum for each group across the study ( ^*∗*^*P* < 0.05). (b) Box plot of the mean area under the curve of PCV-3 load in gilt serum per treatment group. (c) Box plot of mean PCV-3 load in tissues of gilts per group. (d) Box plot of mean viral load in serum from piglets for each group and time point. (e) Box plot of mean viral load in tissues from piglets for each group and time point. (f) Correlation between overall PCV-3 load in serum and tissues from piglets (including piglets from T1 and T2 groups at farrowing and weaning). F, farrowing; W, weaning. Different letters in the box-plots (Figures 2(d) and 2(e)) mean *P* < 0.05.

Gilts from T3 remained nonviremic throughout the study. In T1, PCV-3 viremia was detected as soon as 14 days postinoculation (dpi) (SD 14) in all six gilts (100%). Viremia in the T2 group was first detected at 14 dpi (SD 49) in 3/5 gilts (60%), with the other two gilts showing first qPCR positivity at 21 dpi (SD 56) and 42 dpi (SD 77), respectively. In both groups, from the onset of viremia onward, most of the gilts remained positive until the end of the study. Viremia peaked in the T1 group (5.2 log10 PCV-3 genome copies/mL of serum) at 28 dpi (SD 28) and in the T2 group (5.72 log10 PCV-3 genome copies/mL of serum) at 21 dpi (SD 56) (Figure 2(a)). Mean viremia AUC of gilts from both groups was not significantly different, as shown in Figure 2(b) (T1 group: 6.57 log10 PCV-3 genome copies/mL of serum, equivalent to 4.68 log10 PCV-3 genome copies/mL of serum per day; T2 group: 6.41 log10 PCV-3 genome copies/mL of serum, equivalent to 4.70 log10 PCV-3 genome copies/mL of serum per day).

All pooled tissues from PCV-3 inoculated gilts collected at necropsy were also positive for PCV-3 qPCR, with a mean viral load of 6.61 and 6.62 log10 PCV-3 genome copies/mL of tissue supernatant in T1 and T2 groups, respectively (Figure 2(c)). A lack of correlation between serum viral load at necropsy (SD 91) (3.00 and 3.86 log10 PCV-3 genome copies/mL of serum in T1 and T2 groups, respectively) and tissue viral load in gilts was observed. Viral loads in tissues were 3–3.5 log10 higher than in sera.

### 3.3. Piglets from Gilts Inoculated at the Second Third of Gestation Displayed Higher Percentage of PCV-3 Infection

The pool of tissues from T1 mummies were qPCR positive (2/2; 100%, mean viral load: 5.65 log10 PCV-3 genome copies/mL of tissue supernatant), whereas those from group T2 were all negative (0/3, 0%). For the stillborn, almost all animals from T1 (9/10; 90%) were PCV-3 qPCR positive (mean viral load: 7.13 log10 PCV-3 genome copies/mL of tissue supernatant), while all stillborns from T2 and T3 were negative (0/2 (0%) and 0/3 (0%), respectively). Overall, viral load in mummies was lower than that of born alive and stillborn piglets; however, this difference was not statistically significant. The number and proportion of mummified and/or stillborn piglets did not correlate with the AUC of the gilts nor with their viral load in serum at farrowing or at any other time point (data not shown).

The percentage of viremic piglets and the mean viral load in serum of all piglets within the groups were not statistically different to the subgroup euthanized at farrowing and the one at weaning (Figure S1).

At farrowing, 58/62 (93.5%) of the total born alive piglets were viremic in the T1 group (mean viral load: 5.30 log10 PCV-3 genome copies/mL of serum), which was significantly higher than those of the T2 group (26/48, 54.2%; mean viral load: 3.14 log10 PCV-3 genome copies/mL of serum) (Figure 2(d)). Viral presence and load in tissues from piglets euthanized at farrowing were also significantly higher in the T1 group (32/33; 97.0%, mean viral load: 7.69 log10 PCV-3 genome copies/mL of tissue supernatant) than in the T2 group (19/28; 67.9%, mean viral load: 3.28 log10 PCV-3 genome copies/mL of tissue supernatant) (Figure 2(e)).

Most T1 piglets raised to weaning age (21 days of life approximately) remained viremic at that time point (24/31, 77.4%); although there was a statistically significant decrease in mean viral load in serum (4.54 log10 PCV-3 genome copies/mL serum; Figure 2(d)) compared to farrowing. On the contrary, percentage of viremic piglets from the T2 group increased (19/23, 82.6%) compared to farrowing. Moreover, in this group, the viral load in serum at weaning was significantly higher (4.58 log10 PCV-3 genome copies/mL serum) than at farrowing (Figure 2(d)).

Almost all piglets at weaning from the T1 group yielded positive results in tissues (30/32, 93.8%), and piglets from the T2 group displayed similar percentage of positive results (16/23, 69.6%) than at farrowing. Viral load from tissues from piglets in T1 at weaning was numerically lower (6.89 log10 PCV-3 genome copies/mL of tissue supernatant) compared to farrowing; instead, T2 piglets had numerically higher mean viral load (6.03 log10 PCV-3 genome copies/mL of tissue supernatant) compared to farrowing, although it was not statistically significant (Figure 2(e)).

Overall, the PCV-3 load in serum at moment of piglet necropsy showed a statistically significant correlation with the viral load in pooled tissues (Figure 2(f)). Such correlation was also observed when assessed per group (T1 or T2) or time point (farrowing or weaning) (data not shown).

All samples (serum and pooled tissues from alive, mummies, and stillborn) from T3 group piglets were qPCR negative.

### 3.4. Coincidence of Full-Length Sequences of Virus Inoculum and Virus from Serum and Tissues

To exclude the possibility of an uncontrolled infection with a potential circulating PCV-3 virus, the sequence of cap gene (ORF2) from the inoculum was compared to the amplified sequence from tissue samples from gilts (three per group) and piglets (two per group). In all cases, the obtained PCV-3 sequences in tissues showed 99%–100% of similarity with the inoculum confirming that the PCV-3 detected corresponded to the inoculated virus.

### 3.5. Periarteritis Is the Main Histological Lesion in Piglets Infected Transplacentally with PCV-3

**Figure 3:**
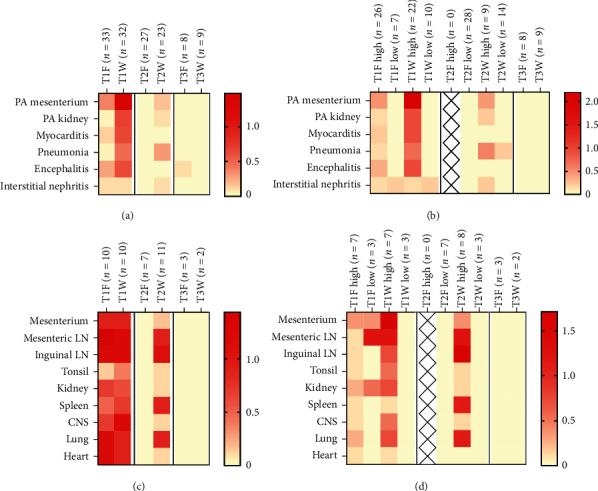
(a) Heatmap representing the mean lesion score of periarteritis (in the mesenterium and kidney), myocarditis, pneumonia, encephalitis, and interstitial nephritis for each group and time point (F, farrowing; W, weaning). Scores have a theoretical maximum of 3; however, the maximum average score was 1.48, which was set as the maximum value in the graphic (bright red). (b) Heatmap representing the mean lesion score for each lesion (maximum value in the graphic: 2.19), for each group and time point, segregated by viral load in tissues (high means >6 log10 genome copies/mL of tissue supernatant); low means ≤ 6 log10 genome copies/mL of tissue supernatant). Negative animals from T1 and T2 groups were excluded, since they did not display lesions. (c) Heatmap representing labeling by ISH within the different tissues, for each group and time point (maximum value in the graphic: 1.44). (d) Heatmap representing labeling by ISH for each group and time point (maximum value in the graphic: 1.71), segregated by viral load in tissues.

Histopathological findings were recorded and graded for all animals. Moreover, ISH was performed in a subset of animals. For the gilt inoculated groups (T1 and T2), a total of seven to eight piglets with high viral load and three piglets with low-intermediate viral load were studied for each time point. No animals in group T2 at farrowing had high viral load; therefore, in this case, seven animals with low-intermediate viral load were evaluated. As negative controls, three animals at farrowing and two animals at weaning from T3 were studied. A summary of recorded lesions is shown in Table 1. Mean scores for lesions are shown in Figure 3(a) (total per group) and Figure 3(b) (segregated by viral load in tissues). Similarly, mean scores for ISH labeling are shown in Figure 3(c) (total per group) and Figure 3(d) (segregated by viral load in tissues).

**Figure 4:**
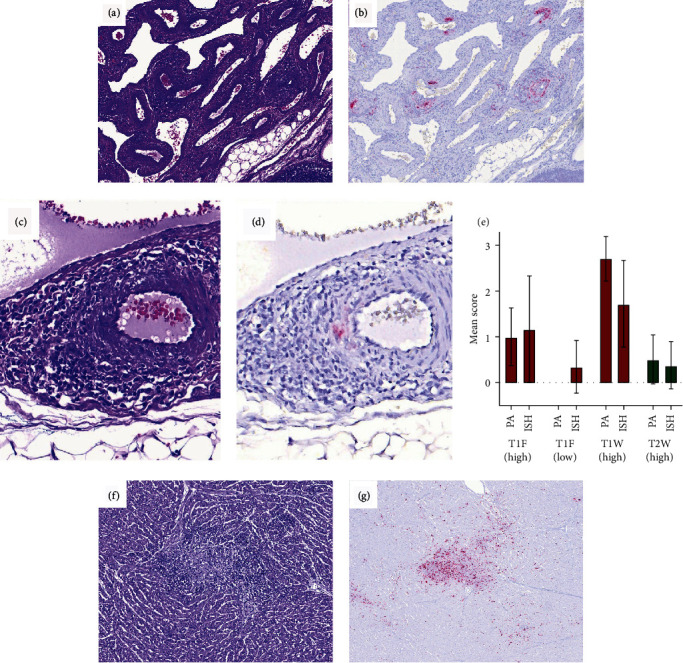
(a) Mesenteric arteries from a piglet at weaning from the T1 group showing multifocal lymphohistiocytic periarteritis, with occasional and segmental involvement of the tunica media (arteritis), score 3 (H&E). (b) PCV-3 ISH labelling of the mesenteric arteries, score 3 (hematoxylin counterstain). (c) Mesenteric artery from a piglet at weaning from T2, showing focal lymphohistiocytic periarteritis and arteritis, score 1 (H&E). (d) PCV-3 ISH labelling from the same animal showing low amount of viral genome in the tunica media, score 1 (hematoxylin counterstain). (e) Mean periarteritis (PA, left columns) and ISH (right columns) scores at the mesenteric arteries in piglets from the T1 group at farrowing (with low and high viral load), and T1 and T2 groups at weaning (high viral load). (f) Heart section revealing focal loss of cardiomyocytes and replacement with loose fibrous tissue with lymphohistiocytic inflammation, score 3 (H&E). (g) PCV-3 ISH labelling of the myocardial section revealing abundant PCV-3 genome, especially in the inflamed area, score 3 (hematoxylin counterstain).

The most frequently found lesion consisted of segmental to circumferential lymphoplasmacytic to lymphohistiocytic inflammation of the tunica adventitia and, occasionally, segmentally the tunica media of the medium and small caliber arteries (lymphohistiocytic periarteritis and arteritis) (Figure 4(a)). Occasionally, vacuolation of the muscular cells in the tunica media and plumping of the endothelium in the tunica intima were seen. Periarteritis was more commonly seen in the mesenterium followed by kidneys; however, its presence was also recorded in other tissues such as spleen, heart, liver, lung (associated to bronchial arteries), and central nervous system (CNS) (in the meningeal vessels) (Table 1).

A high percentage of T1 piglets at weaning displayed periarteritis, followed by T1 piglets at farrowing and T2 piglets at weaning. Moreover, when present, mean periarteritis severity score in the mesenterium was higher in animals from the T1 group at weaning (mean score: 1.48), followed by animals from T1 at farrowing (mean score: 0.38). All animals in T2 at weaning with periarteritis received a score of 1 (mean score: 0.17). No animals from the T2 group at farrowing or from the T3 group displayed periarteritis.

PCV-3 labeling by ISH in arteries matched the presence of periarteritis (Figure 4(b)). When present, labeling was observed in the endothelium, smooth muscle cells, and the adventitial inflammatory infiltrate. Labeling by ISH in mesenteric arteries in piglets with high viral loads was also higher in T1 piglets at weaning (mean score: 1.71), followed by T1 ones at farrowing (mean score: 1.17) and T2 animals at weaning (mean score: 0.38) (Figure 3(d)). However, there was a slight tendency of T1 animals at farrowing to display higher scores of ISH compared to the periarteritis ones. On the contrary, animals at weaning (either from T1 or T2 groups) had higher score of lesions than ISH scores (Figure 4(e)). This latter result is due to occasional animals displaying moderate-to-severe inflammation associated to low or absent labeling (Figures 4(c) and 4(d)), suggesting an eventual viral clearance.

### 3.6. PCV-3 Labeling Was Present in Other Histological Lesions than Periarteritis

Histopathological evaluation of piglets revealed multisystemic inflammation in different tissues. In the heart, lymphohistiocytic myocarditis was seen exclusively in T1 (at farrowing and weaning) piglets associated with PCV-3 labeling (Figures 4(f) and 4(g)). Piglets at weaning had higher lesion score compared to farrowing; however, labeling by ISH was higher at farrowing than at weaning.

Nonsuppurative encephalitis mainly characterized by glial foci and perivascular cuffing was associated with PCV-3 labeling, mainly seen in T1 piglets. A higher number of piglets at weaning had encephalitis, as well as more severity of such lesion and higher score of PCV-3 labeling, when compared to T1 piglets at farrowing. No evidence of encephalitis was observed in T2 animals; only one piglet from this group at weaning displayed mild PCV-3 labeling with no associated lesion. Noteworthy, one animal in T3 at farrowing age displayed mild nonsuppurative encephalitis, probably reflecting a low degree of background lesion in the CNS. No viral genome was detected in any T3 piglet by ISH.

**Figure 5:**
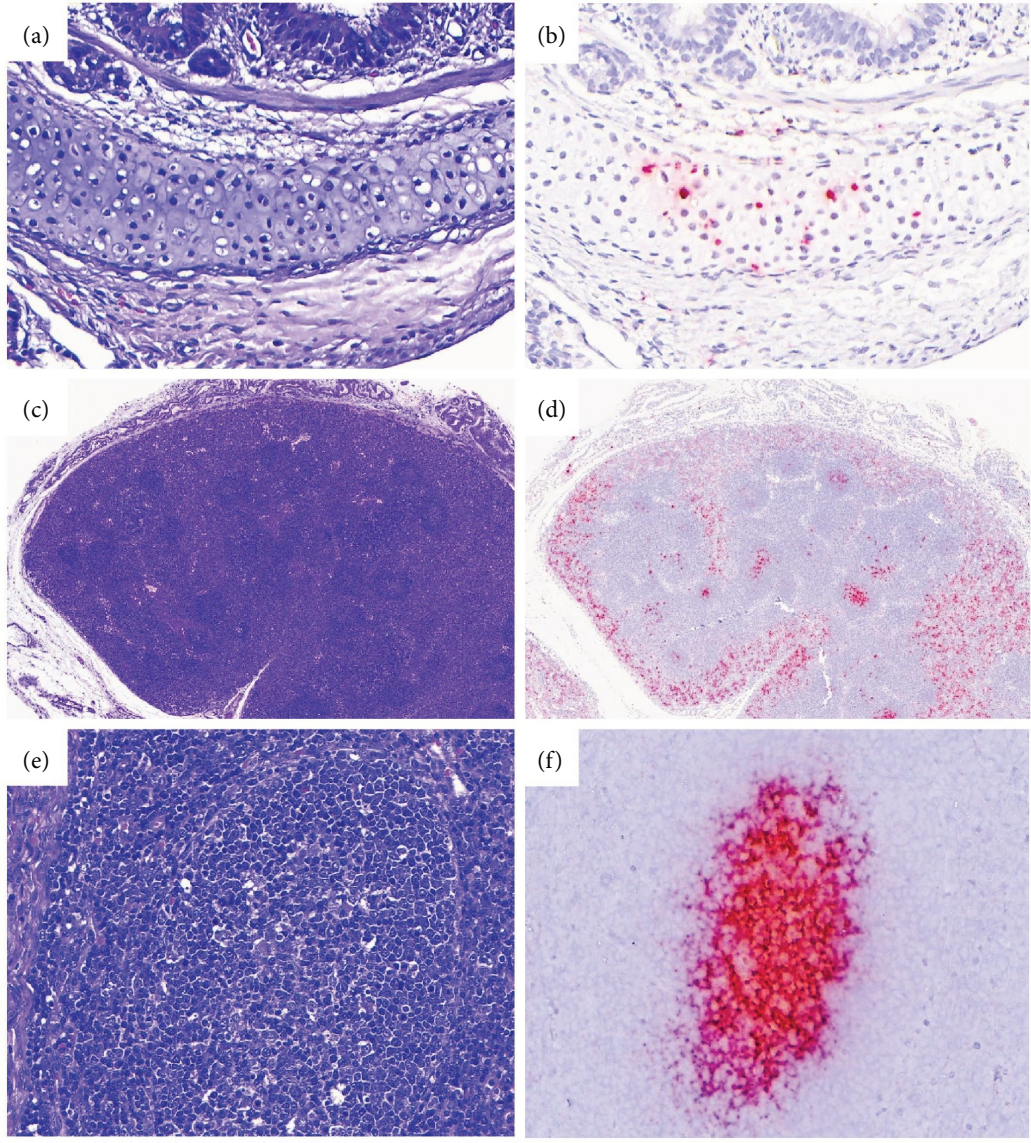
(a) Lung section with histologically normal hyaline cartilage in the bronchi (H&E). (b) PCV-3 ISH of the same section, revealing labelled chondrocytes (hematoxylin counterstain). (c) Histologically normal lymph node (H&E). (d) PCV-3 ISH of the same lymph node, revealing abundant PCV-3 genome within follicles and in parafollicular areas, especially in subcapsular sinuses (hematoxylin counterstain). (e) Prominent lymphoid follicle, histologically normal (H&E). (f) Same lymphoid follicle by PCV-3 ISH, revealing intense positive signal (hematoxylin counterstain).

In the lung, interstitial pneumonia was seen more frequently in animals at weaning (T1 > T2) and rarely at farrowing (only in T1), corresponding with labeling by ISH within alveolar septa. Viral genome amount was higher in animals with high viral load measured by qPCR from group T1 at farrowing, followed by animals at weaning from T1 and T2 groups. Chondrocytes were also frequently labeled in the bronchial cartilage, although not associated with a histological alteration of the chondrocytes (Figures 5(a) and 5(b)).

Interstitial nephritis was recorded in a few cases in animals from the T1 group (at farrowing and weaning) and the T2 group (at weaning); however, lesions had a similar severity (mild) in all these groups and associated with low amount of PCV-3 genome by ISH.

The most common type of ISH labeling observed in lymphoid tissues (mesenteric and inguinal lymph nodes, tonsil, white pulp in the spleen) followed a follicular pattern with no associated histological alteration of the follicles. Labeled follicles revealed activated and prominent germinal centers with large cells at its center with occasional mitotic figures (Figure 5(c)–5(f)), suggesting lymphoid activation. Rarely, parafollicular labeling was recorded. ISH scores in lymphoid tissues were similar between animals with high viral load from T1 (at farrowing and weaning) and T2 (at weaning) groups. Only one animal with low viral load (T1 piglet at farrowing) displayed follicular labeling in the mesenteric lymph node (Figure 3(d)).

No significant histologic alterations were observed in any stillborn or mummified fetus, animals from T3 (aside from the animal showing very mild nonsuppurative encephalitis) nor in the gilts. No labeling by ISH was observed in any of the tissues evaluated from animals at farrowing (T2) and at weaning (T1 and T2) with low viral loads, neither in the negative animals (T3).

### 3.7. PCV-3-Associated Periarteritis Was Associated to Lower Body Weight at Weaning

**Figure 6:**
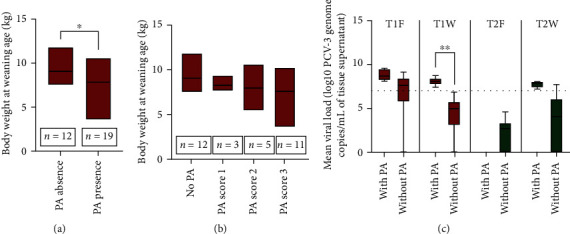
(a) Mean body weight of piglets in group T1 at weaning segregated by presence or absence of periarteritis in the mesenterium ( ^*∗*^*P* < 0.05). (b) Mean body weight of animals in group T1 at weaning segregated by their mean scores of periarteritis in the mesenterium (0–3). (c) Mean viral load in tissues of piglets by group and time point (F, farrowing; W, weaning) segregated by presence or absence of periarteritis in the mesenterium. The horizontal line represents the value of 7 log10 PCV-3 genome copies/mL of tissue supernatant ( ^*∗∗*^*P* < 0.01).

No statistically significant correlation was observed between body weight at farrowing or at weaning with viral load in serum or tissue at farrowing or at weaning. However, piglets from group T1 at weaning with mesenteric periarteritis had significantly lower body weight at weaning (Figure 6(a)) and numerically lower ADWG than those without such lesion (data not shown). When T1 piglets were split by periarteritis scores, animals with higher mesenteric periarteritis scores had less numerical body weight at weaning (Figure 6(b)) and ADWG than those with lower scores; however, this comparison was not significant (data not shown).

There was no correlation between mesenteric periarteritis score and viral load in tissue or viral load in serum (either at farrowing or weaning). However, all animals with mesenteric periarteritis had viral load >7 log10 PCV-3 genome copies/mL of tissue supernatant, being significantly different in animals in T1 at weaning (Figure 6(e)).

### 3.8. Piglets with Periarteritis Were Distributed Evenly Throughout Litters

When assessed by litter, productive parameters (body weight at farrowing and weaning, ADWG) of piglets showed a strong statistical association to their litter. However, animals with presence/absence of mesenteric periarteritis were unevenly distributed in piglets in the T1 group, ranging from 0.0% to 75.0% affected piglets per litter (mean 36.0%). Within the T2 group, only four piglets from one gilt displayed periarteritis (36.4%), while the piglets from other gilts displayed no lesions (mean 7.3%) (Table S2). Overall, presence or absence of periarteritis or its score did not correlate with gilt AUC or its mean viral load at any time point. Body weight at weaning varied greatly throughout litters; however, animals with mesenteric periarteritis within a litter consistently had lower body weight at weaning than those without periarteritis in the same litter (Table S2).

## 4. Discussion

The existing diagnostic criteria for PCV-3-RD and PCV-3-SD comprise compatible clinical signs, characteristic histological lesions (periarteritis), and presence of moderate to abundant viral genome within those lesions [6]. Their difference strives on the fact that the first condition is linked to reproductive disorders, while the second one is mainly characterized by wasting in postnatal pigs. In the present study, piglet pathological findings at farrowing matched the description of PCV-3-RD lesions in fetuses/newborns, while their presence in weaning pigs coincide with those described in the proposed PCV-3-SD case definition. Since the older the pigs, the more severe lesions in both experimental groups, it is tempting to speculate that the so-called PCV-3-SD [6] is probably the reflection of the persistent infection of piglets exposed to PCV-3 as fetuses during gestation. In fact, it has been described under natural infection circumstances that piglets born alive in a litter with signs of PCV-3-RD may grow to weaning age and display signs of systemic disease [22], which would further reinforce the idea that there is not a clear division between PCV-3-RD and PCV-3-SD.

The pathological picture derived from the transplacental infection in the piglets was characterized by multisystemic inflammation. This lesion resembles the ones seen under natural infections both in reproductive [1, 7–9] and postweaning [9–11] cases, in which multisystemic lymphohistiocytic periarteritis is their hallmark lesion. The affected piglets also displayed a range of lesions in other organs (lymphohistiocytic myocarditis, interstitial pneumonia, non-suppurative encephalitis, interstitial nephritis) in conjunction with viral presence by ISH. All these findings emphasize the PCV-3 pathogenic potential as causative for these pathological findings. Altogether, this study represents the first description of PCV-3 transplacental infection by means of an experimental inoculation of pregnant dams at different gestational times and describes the persistent nature of PCV-3 in infected animals.

Interestingly, systemic nonsuppurative periarteritis was observed (in clinically healthy animals) accompanied by a lower body weight (significant at weaning age in piglets from the T1 group). Since PCV-3 has been found to subclinically infect piglets worldwide, the present study suggests that a certain proportion of these infected pigs may have histological lesions leading to a lower productive performance that have remained unknown. This is the first experimental evidence that PCV-3-associated lesions in piglets are linked to a poorer growth, suggesting that even PCV-3 subclinical infection may be relevant to swine industry. In fact, such situation has been already described for PCV-2 infection, the most well-known porcine circovirus [23].

**Figure 7:**
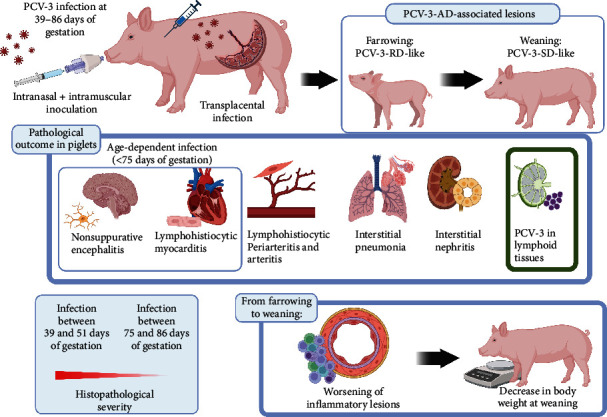
Proposed pathogenesis scheme of the intrauterine infection by PCV-3. The experimentally exposed (intranasally and intramuscularly) pregnant gilts to PCV-3 resulted in transplacental infection of a proportion of fetuses and delivery of PCV-3-infected piglets. These piglets featured PCV-3-associated disease histopathological lesions (as described by Saporiti et al. [6]) at farrowing and at weaning ages, as well as presence of PCV-3 in lymphoid tissues. Infection of cardiac and nervous tissue appeared to be gestational age-dependent, since only piglets coming from gilts inoculated at the second third of gestation (T1) developed myocarditis and encephalitis. From farrowing to weaning, a worsening of these inflammatory lesions was observed, which was accompanied by a decrease in body weight at weaning. Histopathological lesions were always more severe in piglets from gilts inoculated during the second third of gestation (T1), suggesting that the earlier the infection during gestation, the more severe lesions.

At weaning, T1 piglets had more prevalent and severe lesions than animals T2 ones, despite having similar viral loads in serum and tissues. Noteworthy, as evidenced by histological evaluation and ISH, myocardium and CNS displayed lesions and labeling by ISH primarily in animals from the T1 group (at farrowing and at weaning), suggesting a tissue tropism related to gestational age. None of the animals from T2 had myocardial labeling and very rarely displayed labeling within the CNS. These results would imply that PCV-3 is only capable of infecting cardiomyocytes and CNS cells before fetal immunocompetence, and that once these cell types are infected, viral presence in these tissues may be observed at least up to weaning age (Figure 7). Again, these descriptions of PCV-3-SD [1, 9–11, 22] would fit with the results observed in the present study.

The apparent gestational age-related tissue tropism of PCV-3 might not be surprising, since another porcine circovirus (PCV-2) able to cause reproductive and postweaning disorders has shown different cell and tissue targets depending on the age of infection [24, 25]. In fact, porcine circoviruses lack a DNA polymerase, hence they rely on infecting cells that eventually undergo mitosis to successfully replicate. For this reason, we suggest that earlier fetal infection results in replication within cardiomyocytes and CNS cells (presumably neurons and glial cells), resulting in these cell types being persistently infected at least as late as the 21st day of life. Furthermore, frequent infection of chondrocytes in bronchial hyaline cartilage was observed for the first time in this study. We speculate that infection of chondrocytes may also be developmental dependent, considering that chondrocytes are a rather proliferative cell type in early stages of growth (specially in growth plates). In fact, cases of PCV-3-RD and PCV-3-SD in which affected piglets had ear malformations consisting of thrown-back ears [7, 22] have been documented. Unfortunately, presence of PCV-3 in the hyaline cartilage of the ears was not specifically investigated. The possible consequences of PCV-3 infection in hyaline cartilage are yet unknown and deserve future investigation.

Taken all results together, we hypothesize that the earlier the infection of fetuses, the higher susceptibility to replication in cardiac and nervous tissues, which ultimately is the main reason for the disease being more severe in piglets from gilts inoculated earlier during the gestation (Figure 7). The fact that the current experimental infection did not show clinical evidence of reproductive or postweaning disorders should not be surprising, since it already happens with PCV-2 experimental inoculations, a well-established swine pathogen [26, 27]. Interestingly, PCV-2 reproductive disease has been reproduced by inseminating gilts with PCV-2 spiked semen [27, 28], resulting in pregnancy failure or large number of mummified fetuses. At this stage, it is not possible to confirm or rule out whether PCV-3 could infect sows upon artificial insemination, although the presence of virus in semen has been already demonstrated [29]. Obviously, through the present study, we cannot establish the potential outcome of PCV-3 infection during the first third of gestation. However, it can safely be said that inoculation of pregnant dams in the second and last third of gestation may result in an apparently healthy parturition with infected piglets displaying characteristic lesions of both reproductive and postnatal disease. The potential effect of such PCV-3 transplacental infection in combination with other reproductive pathogens is totally unknown at present.

Importantly, fetal immunocompetency has its onset around 70th day of gestation [21]. For this reason, piglets from the T1 group were presumably infected before immunocompetency, while piglets from T2 group got infected after immunocompetency. More severe disease in T1 might suggest that immunotolerance to PCV-3 can play a role in disease outcome. For instance, immunotolerance has been deeply studied in ruminants infected with pestiviruses before fetal immunocompetency, which then suffer from prolonged viremia in absence of cellular and humoral response [30]. Unfortunately, the PCV-3 serological status of these pigs could not be determined due to the absence of validated antibody tests. However, from farrowing to weaning in T1, an enhanced multisystemic inflammation was seen associated to a decrease in tissular viral load and slight decrease of labeling by ISH, which may suggest partial viral clearance by immune system and probably a degree of cellular (and even humoral) immune response. Infected gilts, as well as piglets from group T2, were immunocompetent when infected; however, they remained viremic for almost the entire duration of this study, which further questions whether an immune response with capacity for PCV-3 clearance existed. Overall, the immune response against PCV-3 is largely unknown and deserves further attention in the future.

None of the piglets had lymphocyte depletion nor histiocytic replacement in lymphoid tissues. Although previous reports claimed presence of such lesions in PCV-3-infected pigs [31], viral presence in lymphoid tissue in the piglets in this study was only accompanied by follicular activation. These results match with studies that found no evidence of association between PCV-3 infection and lymphoid depletion or histiocytic replacement [13]. None of the piglets nor gilts in this study displayed clinical signs nor histological lesions compatible with PDNS either.

Based on the information generated in the present study and existing literature, we propose a pathogenic scheme for PCV-3 infection (Figure 7). The transplacental infection and development of lesions in neonatal pigs obtained in the present study are consistent with the so-called PCV-3-RD, while the lesions in older piglets fit with the case definition of PCV-3-SD. Therefore, the present results suggest that PCV-3-RD and PCV-3-SD are probably two phases of the intrauterine PCV-3 infection; hence, we propose that PCV-3-associated disease (PCV-3-AD) as a unique term to express both scenarios.

Last, this study may serve to establish a rational approach to PCV-3-AD diagnosis under field conditions. Since the number of piglets displaying histopathological lesions is variable depending on the infection timing and their age, necropsy and/or sampling multiple piglets per litter should be needed when PCV-3-associated disorders are suspected. Moreover, mummified fetuses had lower viral loads (despite not being statistically significant due to low number of them). For these reasons, the study of stillborn or weak born piglets is probably more efficient than sampling mummies. Finally, a tentative viral load threshold associated to periarteritis can be set at 7 log10 PCV-3 copies/mL of tissue supernatant.

## Figures and Tables

**Table 1:** Proportion and percentage of piglets displaying histopathological lesions in each tissue for each group and time point.

Lesion	T1F	T1W	T2F	T2W	T3F	T3W
PA-mesenterium	10/33 (30.3%)	19/32 (59.4%)	0/27 (0%)	4/23 (17.4%)	0/8 (0%)	0/9 (0%)
PA-kidney	1/33 (3.0%)	13/32 (40.6%)	0/27 (0%)	2/23 (8.7%)	0/8 (0%)	0/9 (0%)
PA-spleen	3/33 (9.1%)	14/32 (43.8%)	0/27 (0%)	3/23 (13.0%)	0/8 (0%)	0/9 (0%)
PA-meninges	0/0 (0%)	12/32 (37.5%)	0/27 (0%)	0/23 (0%)	0/8 (0%)	0/9 (0%)
PA-lung	0/0 (0%)	5/31 (16.1%)	0/27 (0%)	1/23 (4.3%)	0/8 (0%)	0/9 (0%)
PA-heart	0/0 (0%)	6/31 (19.4%)	0/27 (0%)	0/23 (0%)	0/8 (0%)	0/9 (0%)
PA–liver	0/0 (0%)	5/32 (15.6%)	0/27 (0%)	0/23 (0%)	0/8 (0%)	0/9 (0%)
Lymphohistiocytic myocarditis	3/33 (9.1%)	16/31 (51.6%)	0/27 (0%)	0/23 (0%)	0/8 (0%)	0/9 (0%)
Interstitial pneumonia	1/33 (3.0%)	14/31 (45.2%)	0/27 (0%)	6/23 (26.1%)	0/8 (0%)	0/9 (0%)
Nonsuppurative encephalitis	8/33 (24.2%)	18/32 (56.3%)	0/27 (0%)	0/23 (0%)	1/8 (12.5%)	0/9 (0%)
Interstitial nephritis	3/33 (9.1%)	3/32 (9.4%)	0/27 (0%)	2/23 (8.7%)	0/8 (0%)	0/9 (0%)

F, farrowing; W, weaning; PA, periarteritis; T1, piglets from gilts inoculated during the second third of gestation; T2, piglets from gilts inoculated during last third of gestation; T3, piglets from control, non-inoculated gilts.

## Data Availability

The experimental data used to support the findings of this study are available from the corresponding author upon request.

## Supplementary Materials

Supplementary Materials Figure S1: mean viral load in serum from piglets from all animals, segregated by their time point of euthanasia (at farrowing, F, and at weaning, W). Table S1: total born animals (alive, mummified, and stillborn) and available samples for analyses within each group. Table S2: data from individual gilts regarding exact day of gestation at challenge, number of born alive pigs, and percentage of them with or without periarteritis at weaning in respect the body weight.
