# Supplementary material for: Experimental Inoculation of Porcine Circovirus 3 (PCV-3) in Pregnant Gilts Causes PCV-3-Associated Lesions in Newborn Piglets that Persist until Weaning
Source: Transbound Emerg Dis. 2023 Oct 20;2023:5270254. doi: 10.1155/2023/5270254 (PMC12016758; doi:10.1155/2023/5270254)
Supplement: Supplementary Materials — Figure S1: mean viral load in serum from piglets from all animals, segregated by their time point of euthanasia (at farrowing, F, and at weaning, W). Table S1: total born animals (alive, mummified, and stillborn) and available samples for analyses within each group. Table S2: data from individual gilts regarding exact day of gestation at challenge, number of born alive pigs, and percentage of them with or without periarteritis at weaning in respect the body weight. [file 5270254.f1.docx]

# Transboundary and Emerging Diseases

**Experimental inoculation of Porcine circovirus 3 (PCV-3) in pregnant gilts causes PCV-3 associated lesions in newborn piglets that persist until weaning**

Àlex Cobos,^1,2,3,4^ Albert Ruiz,^1,3,5^ Mónica Pérez,^1,3,4^ Anna Llorens,^1,3,4^ Eva Huerta,^1,3,4^ Flor Correa-Fiz,^1,3,4^ Robert Lohse,^6^ Mònica Balasch,^5,*^ Joaquim Segalés,^1,2,3,*^ Marina Sibila,^1,3,4,*^

^1^ Unitat Mixta d’Investigació IRTA-UAB en Sanitat Animal, Centre de Recerca en Sanitat Animal (CReSA), Campus de la Universitat Autònoma de Barcelona (UAB), Bellaterra, 08193 Barcelona, Catalonia, Spain;

^2^ Departament de Sanitat i Anatomia Animals, Facultat de Veterinària, Campus de la Universitat Autònoma de Barcelona (UAB), Bellaterra, 08193 Barcelona, Catalonia, Spain;

^3^ WOAH Collaborating Centre for the Research and Control of Emerging and Re-Emerging Swine Diseases in Europe (IRTA-CReSA), Bellaterra, Barcelona, Spain;

^4^ IRTA Programa de Sanitat Animal, Centre de Recerca en Sanitat Animal (CReSA), Campus de la Universitat Autònoma de Barcelona (UAB), Bellaterra, 08193 Barcelona, Catalonia, Spain;

^5^ Zoetis Manufacturing&Research Spain S.L., Ctra. Camprodon s/n, La Riba, 17813 Vall de Bianya (Girona), Spain;

^6^ Zoetis Inc. 333 Portage St, Kalamazoo, MI 49007, USA.

^*^Equally contributing authors.

Correspondence should be addressed to Marina Sibila; [marina.sibila@irta.cat](mailto:marina.sibila@irta.cat), Joaquim Segalés; [joaquim.segales@irta.cat](mailto:joaquim.segales@irta.cat), Mònica Balasch; [monica.balasch@zoetis.com](mailto:monica.balasch@zoetis.com).

## Supplementary Materials

Supplementary figure 1: Mean viral load in serum from piglets from all animals, segregated by their timepoint of euthanasia (at farrowing, F, and at weaning, W).
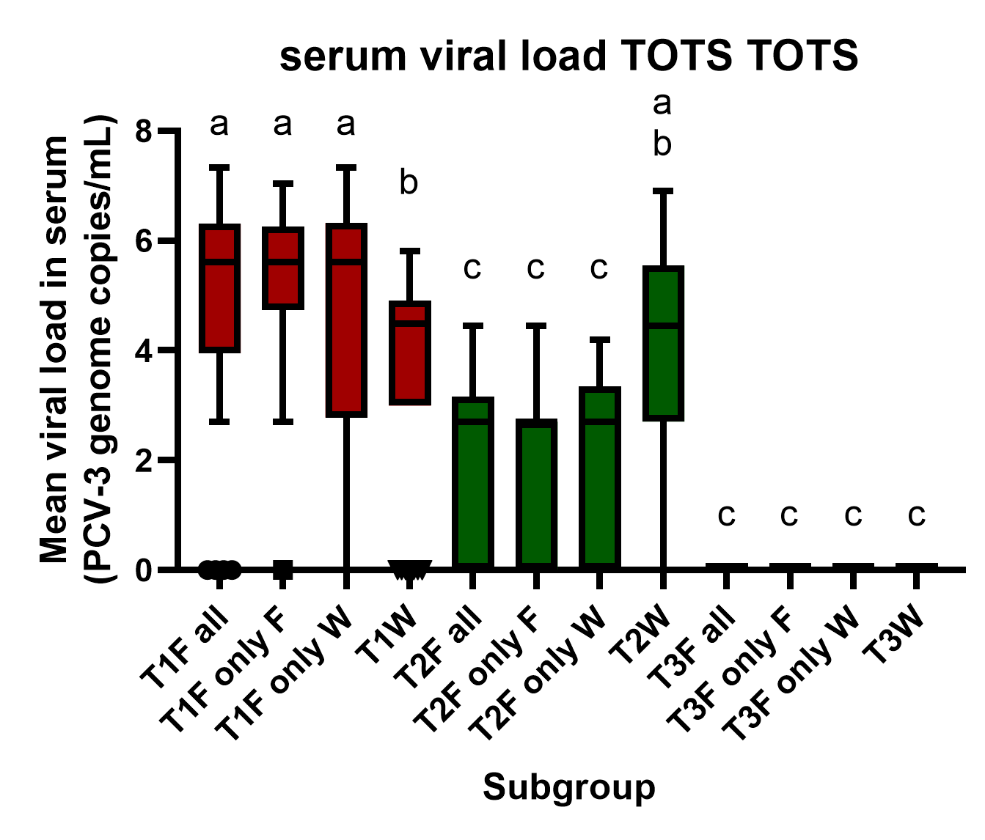


T1F all = all piglets from T1 at farrowing age; T1F only F = piglets from T1 at farrowing, only those euthanized at farrowing age; T1F only W = piglets from T1 at farrowing, only those euthanized at weaning age; T1W = piglets from T1 at weaning age; T2F all = all piglets from T2 at farrowing age; T2F only F = piglets from T2 at farrowing, only those euthanized at farrowing age; T2F only W = piglets from T2 at farrowing, only those euthanized at weaning age; T2W = piglets from T2 at weaning age; T3F all = all piglets from T3 at farrowing age; T3F only F = piglets from T3 at farrowing, only those euthanized at farrowing age; T3F only W = piglets from T3 at farrowing, only those euthanized at weaning age; T3W = piglets from T3 at weaning age;

Supplementary table 1: Total born animals (alive, mummified and stillborn) and available samples for analyses within each group.

|  | Total born | Total mummified | Total stillborn | Total born alive |  | Animals necropsied at F | Serum samples tested at F | Tissue samples tested at F | Animals necropsied at W | Serum samples tested at W | Tissue samples tested at W |
| --- | --- | --- | --- | --- | --- | --- | --- | --- | --- | --- | --- |
| T1 | 77 | 2 | 10 | 65 |  | 33 | 62^1^ | 33 | 32 | 31^2^ | 32 |
| T2 | 56 | 3 | 2 | 51 |  | 28 | 48^1^ | 28 | 23 | 23 | 23 |
| T3 | 20 | 0 | 3 | 17 |  | 8 | 17 | 8 | 9 | 9 | 9 |

T1 = piglets from gilts inoculated during the second third of gestation; T2 = piglets from gilts inoculated during last third of gestation; T3 = piglets from control, non-inoculated gilts; F = farrowing age; W = weaning age; 1In T1 and T2 experimental groups, 3 piglets (each group) were crushed shortly after birth and serum at farrowing age could not be obtained; 2In T1 experimental group, serum from one piglet could not be obtained at weaning age.

Supplementary table 2: Data from individual gilts regarding exact day of gestation at challenge, number of born alive pigs and percentage of them with or without periarteritis at weaning in respect the body weight.

| Group |  | Gilt | Day of challenge | Born alive piglets | Mean BW at W | % piglets with PA at weaning | Mean BW at W (without PA) | Mean BW at W (with PA) |
| --- | --- | --- | --- | --- | --- | --- | --- | --- |
| T1 |  | 4552 | 50^th^ | 10 | 8.51 | 40% | 8.59 | 8.24 |
|  |  | 4624 | 51^st^ | 8 | 10.95 | 33% | 11.32 | 10.22 |
|  |  | 4562 | 48^th^ | 11 | 8.67 | 100% | NA | 8.67 |
|  |  | 4678 | 56^th^ | 11 | 8.90 | 0% | 8.90 | NA |
|  |  | 4560 | 43^rd^ | 10 | 7.44 | 80% | 7.84 | 7.34 |
|  |  | 4575 | 39^th^ | 15 | 7.03 | 86% | 8.00 | 6.86 |
|  |  | Total | 39-56^th^ | 65 | 8.32 | 61% | 9.06 | 7.83 |
| T2 |  | 4531 | 86^th^ | 9 | 8.52 | 100% | NA | 8.52 |
|  |  | 4813 | 83^rd^ | 8 | 9.67 | 0% | 9.67 | NA |
|  |  | 4622 | 81^st^ | 8 | 9.86 | 0% | 9.86 | NA |
|  |  | 4630 | 76^th^ | 15 | 6.94 | 0% | 6.94 | NA |
|  |  | 4679 | 75^th^ | 11 | 5.57 | 0% | 5.57 | NA |
|  |  | Total | 75-86^th^ | 51 | 7.65 | 17% | 7.44 | 8.52 |
| T3 |  | 4671 | NA | 11 | 8.95 | 0% | 8.95 | NA |
|  |  | 4625 | NA | 6 | 7.82 | 0% | 7.82 | NA |
|  |  | Total | NA | 17 | 8.20 | 0% | 8.20 | NA |

T1 = piglets from gilts inoculated during the second third of gestation; T2 = piglets from gilts inoculated during last third of gestation; T3 = piglets from control, non-inoculated gilts. BW = body weight; W = weaning; PA = periarteritis.
